# GPX-Macrophage Expression Atlas: A database for expression profiles of macrophages challenged with a variety of pro-inflammatory, anti-inflammatory, benign and pathogen insults

**DOI:** 10.1186/1471-2164-6-178

**Published:** 2005-12-12

**Authors:** Graeme R Grimes, Stuart Moodie, John S Beattie, Marie Craigon, Paul Dickinson, Thorsten Forster, Andrew D Livingston, Muriel Mewissen, Kevin A Robertson, Alan J Ross, Garwin Sing, Peter Ghazal

**Affiliations:** 1 The Scottish Centre for Genomic Technology and Informatics, University Of Edinburgh, 49 Little France Crescent, Edinburgh, EH16 4SB, UK

## Abstract

**Background:**

Macrophages play an integral role in the host immune system, bridging innate and adaptive immunity. As such, they are finely attuned to extracellular and intracellular stimuli and respond by rapidly initiating multiple signalling cascades with diverse effector functions. The macrophage cell is therefore an experimentally and clinically amenable biological system for the mapping of biological pathways. The goal of the macrophage expression atlas is to systematically investigate the pathway biology and interaction network of macrophages challenged with a variety of insults, in particular via infection and activation with key inflammatory mediators. As an important first step towards this we present a single searchable database resource containing high-throughput macrophage gene expression studies.

**Description:**

The GPX Macrophage Expression Atlas (GPX-MEA) is an online resource for gene expression based studies of a range of macrophage cell types following treatment with pathogens and immune modulators. GPX-MEA follows the MIAME standard and includes an objective quality score with each experiment. It places special emphasis on rigorously capturing the experimental design and enables the searching of expression data from different microarray experiments. Studies may be queried on the basis of experimental parameters, sample information and quality assessment score. The ability to compare the expression values of individual genes across multiple experiments is provided. In addition, the database offers access to experimental annotation and analysis files and includes experiments and raw data previously unavailable to the research community.

**Conclusion:**

GPX-MEA is the first example of a quality scored gene expression database focussed on a macrophage cellular system that allows efficient identification of transcriptional patterns. The resource will provide novel insights into the phenotypic response of macrophages to a variety of benign, inflammatory, and pathogen insults. GPX-MEA is available through the GPX website at .

## Background

Macrophages are one of the most intensively studied cells of the immune system. They have been shown to play a key role in both the innate and adaptive immune response and are capable of responding to a range of signals leading to classical or alternative activation of immune responses which may be classified as innate, cellular, or humoral, [1]. Their function as a mobile unicellular system make them an ideal biological model for taking a systems perspective to pathway analysis, and *in silico *models of the macrophage cell and signalling pathway are beginning to emerge [2]. Clinically, macrophages are increasingly recognised as key determinants of a wide range of diseases including cancer, atherosclerosis, infection, inflammation and auto-immunity. Because of the broad spectrum of biological processes and pathological conditions involving macrophages, studies and data on macrophage biology are highly heterogeneous and dispersed. There is an emerging need to integrate diverse studies and provide highly reliable datasets for systems level analysis in this cell system. At the Scottish Centre for Genomic Technology and Informatics (GTI), we have developed the GPX-Macrophage Expression Atlas (GPX-MEA). The GTI is a founder member of the Integrated Macrophage Biology Group (IMBG), which is a network of institutes with interests in a wide variety of macrophage related research topics, such as inflammation, infectious disease, cancer biology and immunity. Notably, all members of the IMBG have a common interest in elucidating key pathways within the macrophage and the network allows researchers to contribute a variety of biological knowledge and experimental and computational techniques to this area.

GPX-MEA is a gene expression database that stores MIAME-compliant [3] microarray experiments and associated data from a variety of human and murine macrophage cell types following treatment with pathogens and immune modulators. This was accomplished by the collation of macrophage microarray studies from a diverse group of private and public resources followed by validation and curation into a central database. GPX-MEA complements other larger gene expression repositories such as GEO [4] and ArrayExpress [5] by providing focussed datasets and comparative facilities for macrophage biologists, immunologists and those interested in systems biology. This enables the researcher for the first time to rapidly search gene expression values from a number of experiments involving different macrophage types, under diverse conditions within a single resource. Furthermore, GPX-MEA provides a coherent group of annotated raw analysis files that can be used by the computational biologist for systems level analysis.

A major challenge when using microarray data to elucidate molecular pathways is the development of methods for the reliable comparison of results from different studies. This issue is further compounded by the fact that microarray assays are highly sensitive to technical perturbations and the lack of standard units of gene expression. It has been shown that microarray experiments, even using the same platform, can vary greatly between groups and laboratories [6]. In an attempt to address this issue in GPX-MEA, a quality assessment score for each experiment in the database is derived using defined criteria to give users an indication of the underlying quality of the experiment design, quality control and data structures. This score can also be used to retrieve experiments based on their quality requirements using the GPX-MEA web interface.

Here we report on the data curation process, quality assessment, content, search facilities and integration tools that are components of the GPX-MEA.

### Construction and content

#### Construction

GPX-MEA is part of the GPX database system at the Scottish Centre for Genomic Technology and Informatics (GTI, ). The GPX system comprises two MIAME-compliant microarray databases, GPX Repository and GPX Discovery. The data is first curated into GPX Repository, a biochip Laboratory Information Management System developed at the GTI. GPX Repository is designed around a set of web enabled data entry forms that assist in the data capture process ensuring that MIAME-compliant information is obtained.

On completion of curation, experiments are migrated from GPX Repository to GPX Discovery. GPX Discovery has been designed with web interfaces which allow the user to query and retrieve data within GPX-MEA. It also has the additional functionality of being able to retrieve and compare individual probe signal intensity values across experiments.

The underlying database schema of GPX Discovery, maxdSQL-Plus, is an enhanced version of maxdSQL 1.2 (maxd, ). Enhancement to the maxdSQL schema include extensions to store image analysis files and annotations from the NCBI UniGene database [7].

Data within the GPX-MEA is accessed through a web-based user interface developed at the GTI and implemented using Perl and an Apache-mod_perl server. The web application interfaces with an Oracle 9iR2 relational database management system to search and retrieve the experimental data. The schema, scripts, tools and documentation are available at .

#### Data acquisition

GPX-MEA contains gene expression data from microarray experiments conducted on a variety of human and murine macrophage cell types challenged with pro-inflammatory, anti-inflammatory, benign and pathogen insults.

Microarray studies fulfilling these biological criteria were collated from a survey of five major gene expression repositories (GEO [4], ArrayExpress [5], SMD[8], RAD [9] and CIBEX [10]). In addition to experiments submitted to public microarray repositories, GPX-MEA includes experiments and associated data selected from a survey of over 2500 publications in the field of macrophage immunology and infection. This combined approach to data collation was adopted in an attempt to consolidate data which had previously been scattered among a variety of repositories and laboratories.

The investigators of each experiment selected for inclusion in the database were contacted and asked to supply raw image and image analysis files (Affymetrix DAT and CEL files), and any MIAME information that were not reported in the original publication or database entry. Responses were received from eleven of the initial eighteen investigators contacted to supply data and information to GPX-MEA.

In addition to experimental information and data obtained from the public domain, GPX-MEA also contains original unpublished microarray experiments conducted at the GTI. These experiments characterise the gene expression profile of murine bone marrow derived macrophages grown under standard and serum free growth conditions. Information and associated data files for these and other experiments may be retrieved from GPX-MEA using the GPX Discovery web interface.

#### Data curation

Data submitted to the GPX-MEA has followed a manual curation process. At present, subjective evaluation criteria are employed during the quality assessment of microarray data. As a result, expert manual curation was undertaken to ensure the value and biological relevance of information held within the GPX-MEA. Efforts in the future will be directed to methods for the automation of this process.

The curation process follows standard operating procedures that include the assessment of the experiment data against defined criteria and validation of experiment information against MIAME standards.

For this first public release of GPX-MEA, studies conducted using the Affymetrix GeneChip^® ^platform have been preferentially selected since they have widely recognised quality control parameters, use of standardized manufacturing and experimental protocols and familiarity of the platform with the biological and computational users. Future releases of GPX-MEA will contain experiments hybridized using spotted custom array platforms and other proprietary platforms, such as those from Agilent and GE Healthcare.

Before submission to the database, analysis files were all processed using the same software program and normalisation algorithm. Affymetrix DAT files were processed using Affymetrix-Gene Chip Operating System (GCOS). The CHP files were generated from CEL files using Affymetrix Global scaling normalisation to a Target Intensity Value of 100 (TGT-100). In some instances DAT files were unavailable and the original CEL files were used to generate the CHP files. The use of this global normalization algorithm provides a uniform measure which allows a preliminary comparison of expression values across experiments within the databases. Other values that are stored within the database from the CHP files are the detection call (P, M, A) and the p-value of detection.

#### Quality assessment

GPX-MEA contains experiments of varying complexity and experimental design collated from a variety of resources. To take into account the variations in quality and statistical power of experiments a GTI-derived ordinal scale quality assessment score is assigned to each experiment added to the database. The score enables the user to make an informed judgement on the reliability of results obtained from experiments within GPX-MEA and allows for experiment selection based on quality requirements. It also functions to provide a metric for systems biologist interested in integrating data sets from the database.

The quality assessment score ranges from 0 (low) to 28 (high) and is derived from six defined categories each with a 3-point (0, 1, 2) scale. The score is also stored as a percentage of the maximum attainable value within the database. Categories are also weighted by relative importance of the assessment on a 3-point (1, 2, 3) scale. The scoring categories are as follows: use of consistent standard operating procedures (SOPs) [11], sample quality control, the number of biological replicates in the study, and finally, variation levels and data distributions [12-14]. All scoring categories, scoring levels and weightings are pre-determined (Table 1) and can be viewed using the GPX Discovery interface. Categories and assessment tools were selected after extensive discussion between biological investigators, statisticians and bioinformaticians. This process was informed by the GTI's experience in high throughput microarray experimentation and took in consideration published literature[11,12,14-18]. Initially an additional assessment category was used that assigned a score to the biological relevance of the experiment. This criterion was omitted from the final assessment system so that the score was focussed on technical sources of variation in the study. This resulted in a more reproducible scoring methodology.

**Table 1:** Quality Assessment Criteria for GPX-MEA

**Assessment category**	**Tool**	**Weight**	**(Score)Criteria**
Standard Operating Procedures (SOPs)	Experiment description/protocol	2	2 – Standard Operating Procedures have been documented and followed. 1 – Only partial SOPs have been documented applied or followed 0 – SOPs not known or not followed
Sample size	Experiment description/protocol	3	2 – Number of independent biological samples exceeds 5 per group, allowing for reasonably high statistical power in the hypothesis testing. 1- 3–5 independent biological samples per group, allowing to apply minimum level of statistics 0 – 1 or 2 independent biological samples per group (e.g. pilot experiment), reducing the applicability of statistics.
Image quality	Image scan or numerical representation (Affymetrix)	3	2 – No signs of chip defects, background noise (RawQ) within 5 point range of each other. Scaling factor within 1–2 fold range. 1 – Isolated chip defects, RawQ within 5 point range, scaling factor 1–3 0 – Systematic chip defects, saturation, RawQ outside 5 point range, scaling factor greater than 3.
Target sample quality	Electropherogram, UV-Spectrophotometer	1	2 – Presence of 18S and 28S ribosomal subunits with no sign of degradation in any sample (RNA integrity). Absorbance ratio of A260/280 close to 2 (RNA purity) [16]. 1 – Either RNA integrity or purity criteria are met. 0 – Neither integrity nor purity criteria are met, or sample quality assessment was not performed, or no information provided.
Sample level data variation/distribution	Box-and-whisker plots, supporting MA plots if required.	3	2 – Very consistent array medians and Inter-Quartile-Ranges across all samples before normalisation procedures (note: underlying assumption is that any treatments/conditions should not cause differential expression in more than 5–10% of all gene probes on the array.) 1 – Very small number of inconsistent data distributions with assumptions of a correctable difference in array signal intensity met (all genes on array affected, inter-array gene relationships are linear or global differences are expected, e.g. LPS treatment). 0 – Inconsistent median signal levels and spread (IQR) of data throughout the experiment, no biological explanation given.
Gene level data variation	Coefficient of Variation versus Mean Expression plots	2	2 – Coefficient of Variation for majority of genes across all replicates within a sample group is less than 20%. Genes above this CV level are mostly in the lower signal range (due to measurement error). 1 – A small proportion of all genes has higher CV values than 20%, with a small number of these in the medium to high expression range. 0 – A large proportion of genes has a CV above 20% across most of the expression range.

A quality control assessment is made for each experiment curated into GPX-MEA. The quality assessment score is calculated using quantitative and qualitative scores in different areas; use of Standard Operating Procedures, the number and use of independent biological replicates, quality of image, sample quality control and structure of numeric data (spread, reproducibility). A combination of the different areas is used to give a total quality assessment score. This score can be used to indicate the quality of the underlying experiment in the database and as such is subject to interpretation. Where data is unavailable for assessment no score is assigned.

It is worth noting that given the complexity and variety of experimental designs and data structures, it is not feasible to develop fully objective criteria based on non-arbitrary threshold values, instead we have sought to provide the user with a graded indication of the underlying quality of an experiment with a scoring system that is reproducible.

The first step in scoring an experiment is to obtain MIAME compliant information about an experiment's design and implementation. Using this information the score for SOPs, sample size and sample quality control can be assigned. The next criterion assesses image files and includes analysis of the background noise (RAWQ) and variation in mean signal intensity (Scaling Factor) for the arrays. The final criterion seeks to measure sample level variation and gene level variation from visualizations output using R and Bioconductor[19] software from the raw image analysis files. Scores are assigned to each experiment by the curator based on the criteria in Table 1.

To illustrate the assessment method the scoring of the category "sample size" is described. In the category "sample size", the number of biological replicates per group is determined and scored using the criteria in table 1. In the case of an experiment with 3 biological replicates per group a mid score of 1 is assigned. After the initial score is calculated, it is adjusted by relative importance using the weighting of the category. The sample size category is deemed of high importance[15,17,18] and is given a weighting of 3, therefore the initial score of 1 is multiplied by the category weighting 3, and assigned a value of 3.

A record detailing the score in each category for an individual experiment can be viewed using GPX-MEA. Further, the combined overall percentage score can be used to select and retrieve experiments using the GPX Discovery interface.

Future versions of GPX-MEA will incorporate non-Affymetrix experiments. This will require modifications to the scoring system which are likely to include; more stringent requirements for the numbers of biological replicates used in custom arrays based experiments, due to the larger noise variation, the use of different visualisation techniques to assess quality and an additional criterion to address the suitability of the reference sample.

#### Data content

The current release of GPX-MEA (May 2005) consists of 11 public microarray experiments with a total of 152 hybridizations from 3 Affymetrix GeneChip^® ^platforms (Table 2). Eight of the eleven experiments have an associated literature publication [20-26]. These experiments include the treatment of human or murine macrophages with a range of agents including viral infection (e.g. murine cytomegalovirus), treatment with cytokines (e.g. IFN-alpha, IFN-beta, IFN-gamma, IL-4, IL-6) and serum withdrawal. In addition, the effects of these treatments had been examined using a range of macrophage genotypes in the mouse (wild type, PIAS-/-, IRF1-/-, SOCS3-/-).

**Table 2:** Summary of microarray experiment in GPX-MEA.

**Accession**	**Experimental Factors**	**Host Species**	**Cell types**	**Hybridisations Count**	**Array Type**	**Reference**
GPX-000032	time, compound, genotype	Mouse	BMDMs	17	MG_U74Av2	[20]
GPX-000034	time, infection, compound	Mouse	BMDMs	12	MG_U74Av2	[21]
GPX-000035	compound, genotype	Mouse	BMDMs	12	MG_U74Av2	[21]
GPX-000036	infection, cell type	Human	Dendritic cells, monocyte derived macrophages	28	HG_U95Av2	[26]
GPX-000037	compound, protocol	Human	PBMCs	12	HG_U95Av2	[24]
GPX-000038	compound, protocol	Human	PBMCs	18	HG_U95Av2	N/A
GPX-000039	time, compound	Mouse	RAW264.7	3	MOE430A	[25]
GPX-000040	time, compound, genotype	Mouse	BMDMs	10	MG_U74Av2	[23]
GPX-000041	infection	Mouse	Monocyte derived macrophages	30	MG_U74Av2	[22]
GPX-000043	protocol	Mouse	BMDMs	6	MG_U74Av2	N/A
GPX-000045	protocol	Mouse	BMDMs	4	MG_U74Av2	N/A

BMDMs indicate cells are bone marrow derived macrophages. PBMCs indicate cells are peripheral blood mononuclear cells. N/A indicates experiments unpublished.

At present, GPX-MEA has a further 12 MIAME-compliant experiments with a total of 246 hybridizations stored in a restricted access site. This allows the accommodation of unpublished microarray experiments. Experiments within this access controlled site will be made publicly accessible following scientific publication. Further macrophage gene expression studies have been identified for deposition in the GPX-MEA database and routine updates to the database are scheduled to include emerging research findings.

### Utility

#### Search pages

Users can browse and retrieve data from GPX-MEA using the user oriented GPX Discovery web based graphical interface . Searches can be performed in three different sections of the interface; experiments, arrays and probes. A search is made using a number of separate criteria such as accession number, technology type, target organism, quality assessment score or keywords. For example, all microarray experiments conducted using humans as the source organism can be retrieved by performing a search selecting Homo sapiens from the organism search field. The results of a query are displayed in a table with hyperlinks to the corresponding experiment, array or probe record.

#### Experiment

Experiments are defined in GPX-MEA as the complete set of bioassays (hybridizations) and their descriptions for a common purpose, following Microarray Gene Expression Data Society (MGED) ontology specifications. Once an experiment has been selected the corresponding database entry is displayed. An experiment record shows the provenance, experimental design, protocols, sample information, array details, quality assessment and a list of array measurements. Expression values can be selected from the experiment record by selecting the desired measurement records. Measurement data are presented to the users as a comma separated value (CSV) file. Significantly, the user can also obtain original image analysis files, e.g. the Affymetrix CEL files, from the database providing additional flexibility for an extended analysis. Access to the original image analysis files is an especially valuable resource for those interested in computational analysis and data integration.

#### Array type

The array type section allows the user to browse the list of array types stored within the database. An array type record displays the description of the array type and the manufacturer. The array layout with probe annotation obtained from a local copy of the UniGene database can also be downloaded as a CSV file from a link within a record.

#### Probe

**Figure 1:**
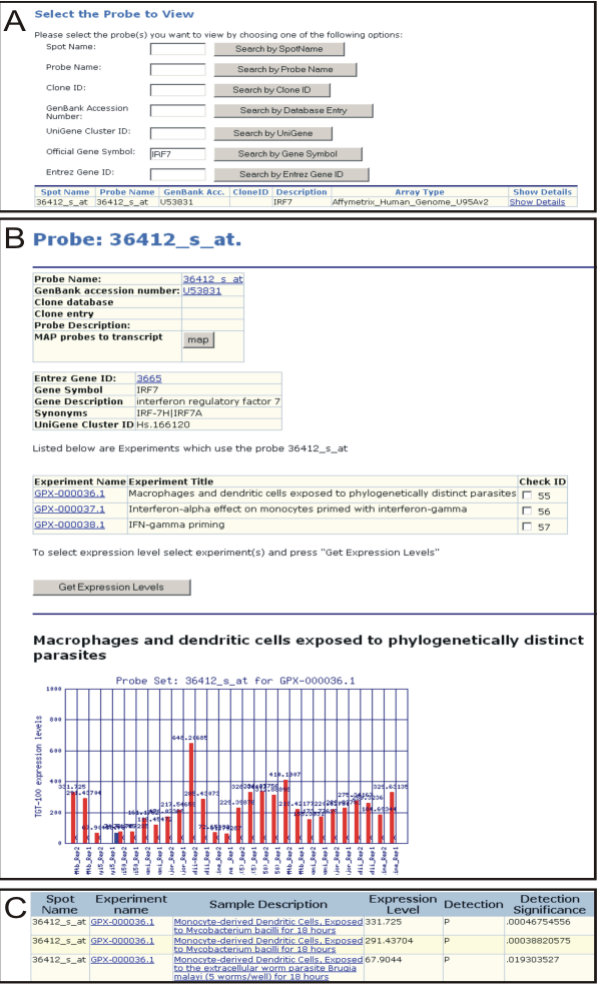
**Comparison of the expression profile of IRF7 across three experiments within GPX-MEA. **(A) From the probe search interface the user searches for the gene 'IRF7'. (B) From the probe record the user selects the experiments they wish to view expression values from. (C) A list of expression values for the probe is displayed alongside the measurement and experiment name.

The probe section allows the user to search and retrieve individual probe details from the database based on criteria such as Entrez Gene ID, Gene Symbol and UniGene ID. Once a probe record is selected the user can view probe annotation within the record and by following links to external databases, Adapt[27], NetAffx [28] and NCBI Gene[29]. The probe record also displays a list of experiments associated with the probe. Using the list of associated experiments, the user can compare the gene expression levels for that probe across the GPX-MEA. For example (Fig 1), the user searches for probes that target the gene interferon regulatory factor 7 using the gene symbol 'IRF7'. From the returned results the user selects a probe record to view more details. From this probe record, the user then selects the experiments within the database to compare the gene expression levels. The results are formatted as a table containing signal intensities, Affymetrix detection calls (P,M,A) and p-value of detections for every measurement from the selected experiments. The signal intensity of Affymetrix probe sets are the TGT-100 normalised signal. The use of this consistent global normalisation algorithm within the database allows for a preliminary comparison of probe expression levels between the distinct microarray experiments.

#### Biolink MatchMaker

The Probe section provides a link to the BioLink MatchMaker integration tool which enables the comparison of different array types. BioLink MatchMaker uses NCBI's UniGene Cluster IDs to identify probes which represent equivalent genes on two different microarray platforms of the same species stored within GPX-MEA. Users can also submit a list of up to 1000 GenBank or UniGene ID to identify probe matches with any array type in the database using the Batch query function. Results can be displayed either as HTML tables containing hyperlinks to the corresponding UniGene entry or can be downloaded as a CSV file for further analysis. For example, the Affymetrix GeneChips^® ^MG U74Av2 and MOE430A version 2 can be compared using BioLink MatchMaker to find probes that share equivalent genes. The results show that there are 7454 unique probe matches between the two platforms. The user can then view the results in a separate webpage or download the results for further analysis.

#### Future work

GPX-MEA is under active development and will continue to collate macrophage expression studies through online database searches and collaboration with principle investigators. This data will be assessed and manually curated within the GPX-MEA and released in routine database updates. In addition, experiments that profile the macrophage response to infection and immune regulators conducted within GTI will also be included in the database. There will also be continual development of curation and quality assessment protocols to maintain the high standard of experimental description and data within the database.

New analytical tools in development for the GPX-MEA include the ability to query gene expression using interactive immunological pathway maps and an automated statistical analysis tool to guide the user through normalisation and analysis processes. The export of MAGE-ML (Microarray Gene Expression-Markup language) from the database is also being developed so that data may be shared between other public repositories and users of microarray data. In addition, GPX is part of the Edinburgh National Translational Cancer Research Network Centre resources (NTRAC) and relevant work emerging from this project will be included in GPX-MEA.

## Conclusion

Here we present GPX-MEA, a MIAME compliant online database that exploits and captures the wealth of expression studies related to macrophage biology data in a centralised resource. Experiments are assessed and submitted to the database by expert curators and includes some information and data, such as image analysis files, that were not previously publicly available.

This is the first example of a focussed macrophage gene expression database that allows efficient identification of gene expression patterns in this key player in the host immune system.

GPX-MEA also provides highly reliable datasets with associated contextual information that can be used by the computational biologist for systems level analysis. In addition, it also utilises a unique quality assessment scoring system that enables the user to select experiments based on their quality requirements.

## Availability

GPX-MEA is publicly available and can be accessed at  using web browsers. The GPX Discovery software is available for download at .

## Authors' contributions

GG carried out the data collation, curation, and quality assessment and drafted the manuscript. SM and GG carried out the database development, design and data migration. TF, KR, PD, MC participated in the design of the quality control criteria. GS, AL, MC, AR contributed data to the database. PG and JB conceived of the database, and participated in its design and coordination. PG, JB, TF, KR, PD, SM and MM helped to draft the manuscript. All authors read and approved the final manuscript.
